# Surgical Scar Endometriosis: An Emerging Enigma

**DOI:** 10.7759/cureus.35089

**Published:** 2023-02-16

**Authors:** Anitha Durairaj, Harini Sivamani, Mahalakshmi Panneerselvam

**Affiliations:** 1 Obstetrics and Gynaecology, Velammal Medical College Hospital and Research Institute, Madurai, IND

**Keywords:** cross-sectional study, lesion characteristics, demographic characteristics, surgical management, surgical scar endometriosis, cesarean section

## Abstract

Introduction

Surgical scar endometriosis is a subtype of extra-pelvic endometriosis that is characterized by the formation of endometrial tissue near the incision site in patients who have previously undergone surgery. In recent times, with the increasing trend in Caesarean sections, the incidence of surgical scar endometriosis has also emerged. This study aims to describe the clinical characteristics and management of surgical scar endometriosis.

Methodology

We conducted this cross-sectional, observational study over eight years (2015-2022) in a tertiary care centre in Madurai district, Tamil Nadu, India. We conducted this study after acquiring an ethical certificate from the institutional ethics committee (IEC No. VMCIEC/22/2018). In this study, we sampled all women (n = 32) with a pathological diagnosis of scar endometriosis during the study period from hospital records (universal sampling). We searched the data for both general characteristics and lesion characteristics of the patients. The general characteristics include age, body mass index (BMI), parity, mode of delivery, symptoms, and imaging by ultrasound. We have recorded the lesion characteristics of the patient, including location and size of scar endometriosis, layers involved in scar endometriosis, and surgical technique from surgical notes written in the case sheet. The minimum sample size required for this study was 31 study subjects. We entered the data into Excel (Microsoft, Redmond, WA, USA) and analyzed it in SPSS version 21 (IBM Corp., Armonk, NY, USA). We expressed the quantitative variables in terms of mean and standard deviation and the qualitative variables in terms of frequency and percentage.

Results

The mean age of the study participants was 34 years (range 23-55 years). In our study, 29 patients (90.6%) were multi-para, and only three (9.4%) were nullipara. Among 29 parous women, the majority (25, or 77.7%) had delivered by Caesarean section, while only four (12.5%) delivered by normal vaginal delivery. The surgical procedures preceding the scar endometriosis were predominantly obstetric procedures (87.4%), out of which 25 patients underwent a Caesarean section and only three underwent an episiotomy. The most common presenting symptom of scar endometriosis in our study was cyclical pain in the scar site (90.4%), followed by swelling (81.25%). In 62.5% of patients, the duration between the presentation of surgical scar endometriosis and surgical intervention was greater than one year. Subcutaneous tissue (90.6%) was the most commonly involved layer in surgical scar endometriosis, followed by the rectus sheath (86.2%). The surgical procedure done for scar endometriosis was wide local excision in 78% of patients, and the remaining 22% of patients had wide local excision with mesh repair.

Conclusion

Cesarean section is an obvious risk factor for surgical scar endometriosis. Clinicians should have a high index of suspicion for surgical scar endometriosis in women presenting with cyclic pain at the scar site. Ultrasound is accurate in diagnosing scar endometriosis. Surgical management by wide local excision with a clear margin with or without mesh repair is the treatment of choice.

## Introduction

Endometriosis is a benign gynecological disorder characterized by the growth of endometrial glands and stroma outside the uterine cavity. Endometriosis is an estrogen-dependent chronic inflammatory disease that can affect either the pelvic or extra-pelvic regions of a woman’s body. We classify it as either pelvic or extra-pelvic endometriosis [1].

Endometriosis is a common condition that affects 5-10% of all women and can cause severe discomfort as well as infertility. It is estimated that endometriosis affects 89 million women of reproductive age around the world [2].

When endometriotic implants are found in areas of the body that are not associated with the pelvic organs, we refer this condition to as having extra pelvic endometriosis. These locations include the gastrointestinal tract, the urinary tract, the lungs, the abdominal wall, and the central nervous system.

Surgical scar endometriosis, also known as SSE, is a subtype of extra-pelvic endometriosis that is characterized by the formation of endometrial tissue near the incision site in patients who have previously undergone surgery. The researchers have described only a few examples of surgical scar endometriosis in the medical literature, making this a rare clinical condition. There have been reports of scar endometriosis developing after obstetric and gynecological surgeries, such as Cesarean sections, vaginal deliveries in episiotomy sites, laparotomies or laparoscopic port sites for hysterectomy, tubectomy, ectopic pregnancies, ovarian cystectomies, hernial repair sites, and even needle tracked after amniocentesis [3,4]. This iatrogenic result is still a mystery, and the exact etiology of surgical scar endometriosis is unknown; there are several hypotheses that attempt to explain its development. There is a wide range of variation in the incidence of surgical scar endometriosis, ranging from 0.03% to 1.08% [5,6].

Due to the unusual presentation of these patients, general physicians, surgeons, or dermatologists may be their initial point of contact. Because of this, it is imperative that medical professionals have more education regarding this disorder [7]. In recent times, with the increasing trend of Caesarean sections, the incidence of SSE has also emerged. This study aims to describe the clinical characteristics and management of surgical scar endometriosis.

## Materials and methods

Study design, duration, and ethical clearance

We conducted this cross-sectional, observational study over eight years (2015-2022) in a tertiary care centre in Madurai district, Tamil Nadu, after acquiring an ethical certificate from the institutional ethics committee of Velammal Medical College Hospital and Research Institute, Madurai (IEC No. VMCIEC/22/2018).

Study sampling and data collection

In this study, we sampled all women (n = 32) with a pathological diagnosis of scar endometriosis during the study period from hospital records (universal sampling). We searched the data for both general characteristics and lesion characteristics of the patients. The general characteristics include age, body mass index (BMI), parity, mode of delivery, symptoms, the duration between index surgery and the onset of symptoms, and imaging by ultrasound (diagnosis). We have recorded the lesion characteristics of the patient, including location and size of scar endometriosis, layers involved in scar endometriosis, and surgical technique from surgical notes written in the case sheet.

Sample size

The minimum sample size required for this study was 31 study subjects. We calculated the sample size by using the formula 3.84*p*q/d^2^, where p is prevalence, q is the complement of p, and d is absolute precision (which was 5%). We collected the prevalence from the study by Yuan et al. in China, where they conclude that the prevalence of abdominal wall endometriosis is 1.96% [8].

Statistical analysis

We entered the data into Excel (Microsoft, Redmond, WA, USA) and analyzed it in Statistical Package for Social Sciences (SPSS) version 21 (IBM Corp., Armonk, NY, USA). We expressed the quantitative variables in terms of mean and standard deviation, and we expressed the qualitative variables in terms of frequency and percentage. The current study was an exploratory one, and we did not assign an outcome variable.

## Results

We included about 32 patients with a pathological diagnosis of surgical scar endometriosis in our study, and we analyzed their results. Table 1 shows the general characteristics of patients with surgical scar endometriosis. The mean age of the study participants was 34 years (range 23-55 years). The mean BMI in our study was 29.15, of which 43.8% were obese. In our study, 29 patients (90.6%) were multi-para, and only three (9.4%) were nullipara. Among 29 parous women, the majority (25, or 77.7%) had delivered by Caesarean section, while only four (12.5%) delivered by normal vaginal delivery.

**Table 1:** General characteristics of the study participants (n=32) BMI – Body Mass Index, LSCS – Lower Segment Cesarean Section

General characteristics	Frequency	Percent
Age (in years)	Mean – 34.47 standard deviation – 8.39	
BMI (kg/m^2^)	Mean – 29.15 standard deviation – 4.72	
Underweight (18)	0	0
Normal (18.5 – 24.9)	7	21.8
Overweight (25 – 29.9)	11	34.4
Obese >30	14	43.8
Parity		
Nullipara	3	9.4
Multipara	29	90.6
Mode of delivery		
Normal vaginal delivery	4	12.5
1 LSCS	8	25.0
2 LSCS	17	52.5

Table 2 shows the lesion characteristics of patients with surgical scar endometriosis. The surgical procedures preceding the scar endometriosis were predominantly obstetric procedures (87.4%), out of which 25 patients underwent a Caesarean section and only three underwent an episiotomy. Two of the procedures preceding scar endometriosis were hysterectomy and endometriotic cyst excision. The mean interval between index surgery and clinical presentation of surgical scar endometriosis was 6.19 years. The most common presenting symptom of scar endometriosis in our study was a cyclical pain in the scar site (90.4%), followed by swelling (81.25%).

**Table 2:** Lesion characteristics and symptoms of the study participants (n=32) LSCS – Lower Segment Cesarean Section

Lesion characteristics		Frequency	Percent
Index surgery			
Episiotomy		3	9.4
LSCS		25	78.0
Hysterectomy		2	6.3
Laparoscopic cyst excision for endometriosis		2	6.3
Duration between index surgery and onset of symptoms (in years)		Mean – 6.19 Standard deviation – 4.25	
Symptoms			
Cyclical pain at scar		29	90.4
Swelling or lump		26	81.25
Cyclical bleeding from lump		4	12.5
Dysmenorrhea		21	65.6
Ultrasound diagnosis of scar endometriosis			
Correct		31	96.8
Wrong		1	3.2
Preoperative medical management		11	34.4
Duration between onset of symptom and surgery			
Within 1 year		12	37.5
More than 1 year		23	62.5
Location of scar endometriosis			
Abdominal wall scar			
I	Suprapubic transverse scar		
	i) Left lateral	17	52.5
	ii) midline	5	15.5
	iii) Right lateral	5	15.5
II	Port site scar		
	i) Left Lateral	1	3.1
	ii) Umbilical	1	3.1
Episiotomy scar		3	9.4
Size of scar endometriosis			
Layers involved in scar endometriosis			
Skin involvement		12/32	37.5
Subcutaneous tissue		29/32	90.6
Rectus sheath		25/29	86.2
Rectus muscle		4 /29	13.7
Peritoneum		12/29	3.1
Perineal muscle		1/3	3.1
Surgical Procedure			
Wide local excision		25	78.0
Wide local excision with mesh repair		7	22.0

Preoperative imaging included ultrasonography in all patients, which is accurate in 96.8% of cases. 34.4% of patients had tried medical management of scar endometriosis before definitive surgical intervention. In 62.5% of patients, the duration between the presentation of surgical scar endometriosis and surgical intervention was greater than one year. In our study we reported the location of surgical scar endometriosis in three sites, namely abdominal wall suprapubic transverse scar in 27 patients (83.5%), abdominal wall port scar in two patients (9.4%), and perineal episiotomy scar in three patients (9.4%). The mean size of the surgical scar endometriosis lesion was 4.5 x 3.5 cm. Subcutaneous tissue (90.6%) was the most commonly involved layer in surgical scar endometriosis, followed by the rectus sheath (86.2%). The surgical procedure done for scar endometriosis was wide local excision in 78% of patients, and the remaining 22% of patients had wide local excision with mesh repair.

## Discussion

Surgical scar endometriosis remains an enigma. The exact cause and natural progression of endometriosis are yet to be determined. Surgeons transplant directly active endometrial cells onto the layers of a surgically incised lesion, and these cells defy immune-mediated apoptosis, allowing an ectopic endometrial cell to survive [9,10]. The estrogen-dependent inflammatory response theory explains the natural progression of endometriosis and its symptoms. We analyzed this emerging iatrogenic complication in the present study.

The mean age of the women with surgical scar endometriosis in our study was 34 years. A study by Yildirim et al. [11] and Zhang et al. [12] also reported a mean age of 31 and 34 years, respectively. We attributed this high prevalence among women of childbearing age to an increase in surgical scar endometriosis patients following Caesarean sections. In our study, patients presenting beyond the reproductive age group were following gynecological procedures. The mean BMI in our study was 29.15, among which 43.8% were obese, which suggests an increased prevalence of scar endometriosis in obese individuals. This finding was similar to the results of the study conducted by Sumathy et al. [13] and Ding et al. [14]. Obesity can provide a wide surgical surface for the entrapment of active endometrial cells and may start the process [10].

Among 29 parous women with surgical scar endometriosis, the majority (25, or 77.7%) had delivered by Caesarean section and only four (12.5%) had delivered by normal vaginal delivery. The index surgery is the one that results in the development or occurrence of endometriosis at the surgical scar site, and we thought it to be an obvious risk factor for surgical scar endometriosis. The index surgeries preceding the scar endometriosis in our study were predominantly obstetric procedures (87.4%), out of which 25 patients were following a Caesarean section. We considered scar endometriosis to increase with parity because of more adhesions and non-closure of peritoneal layers, exposing endometrial cells to the abdominal cavity [15]. Only three women in our study had surgical scar endometriosis after episiotomy, which is because of shedding decidual endometrial tissue implants on the episiotomy site [16]. In our study, four women developed surgical scar endometriosis following gynecological procedures, of which two underwent laparoscopic endometriotic cystectomy and two underwent abdominal hysterectomy. This could be because of the inoculation of endometrial cells onto the incision site while removing the specimen.

The mean interval between index surgery and the clinical presentation of surgical scar endometriosis was 6.19 years (range 2-10 years). Scar endometriosis has a sluggish onset, initially manifesting as cyclical discomfort that is frequently misdiagnosed as dysmenorrhea. The patient usually presents when there is the development of a lump at the scar site.

The most common presenting symptoms of surgical scar endometriosis were cyclical pain, followed by a lump at the scar site. In our study, we observed a cyclical pain, a characteristic symptom of scar endometriosis, in 90% of participants. We found similar findings in a study conducted by Zhang et al. [12] and Buscemi et al. [17]. Cyclical hemorrhage of functioning endometrium in response to hormonal changes is the reason for cyclic pain at the scar site. Cyclical bleeding from the scar site is the specific feature of surgical scar endometriosis, but we report it in only 12.5% of the patients in our study.

In our study, we found surgical scar endometriosis in three locations: the abdominal wall suprapubic transverse scar, the abdominal wall laparoscopic port site, and the episiotomy scar. Among the 27 cases of abdominal wall suprapubic transverse scar endometriosis, 25 were following Caesarean sections, and only two were following abdominal hysterectomy. Two cases of abdominal wall port site endometriosis were following laparoscopic endometriotic cyst excision, and three cases of episiotomy scar endometriosis were following vaginal delivery. The mean size of the surgical scar endometriosis lesion was 4.5 by 3.5 centimeters. Subcutaneous tissue involvement (90.6%), followed by involvement of the rectus sheath (86.2%), was the most prevalent pattern of involvement in surgical scar endometriosis. The study conducted by Sumathy et al. showed 100% of the individuals had subcutaneous tissue involvement and 75% had rectus sheath involvement [13].

We only used imaging as a supplement to a high index of clinical suspicion for diagnosing SSE. Ultrasonography was accurate in 96.8% of patients with scar endometriosis in our study. This finding is comparable to those by Yuan et al. [8] and Zhang et al. [12]. The sonographic appearance of scar endometriosis is predominantly hypoechoic and heterogeneous, with scattered internal echoes at the surgical scar site with limited vascularity [18,19]. Ultrasound imaging eliminates differential diagnoses such as suture granuloma, hematoma, neuroma, hernia, and neoplasia of surgical scar endometriosis, which not only helps in making an accurate diagnosis but also aids in presurgical mapping [20].

The treatment options for surgical scar endometriosis are medical therapy and surgical interventions. Medical therapy with oral contraceptive pills (OCPs), gonadotropin-releasing hormone (GnRH), and danazol is usually futile, providing only temporary symptomatic relief that will recur at a later date. In our study, 34.4% of patients had tried medical management of scar endometriosis before definitive surgical intervention. Hence, surgical intervention is the definitive treatment. We should consider abdominal wall reconstruction with mesh besides wide local excision in larger lesions involving the rectus sheath or muscle that leave a wide post-excisional defect [13]. Failure to do so might cause an incisional hernia, as the approximation of layers is under tension and weak. Complete resection with a 1 cm clear margin and avoiding contamination while handling is critical to preventing recurrence after surgery.

The complications of surgical scar endometriosis are recurrence and malignant transformation. A study conducted by Ding and Zhu reported a recurrence rate of 1.5% [14]. The key to preventing SSE recurrence after surgery is complete resection with a 1 cm clear margin and avoiding endometriotic cell contamination of the field while handling. Transformation of SSE is multifactorial, involving genetic, immunological, and environmental factors. Clear cell carcinoma is the most common histological subtype, followed by endometrial carcinoma [21].

Prevention of surgical scar endometriosis is an absolute need. We must keep the contamination of the surgical incision layers with decidual or endometrial cells to a minimum in order to accomplish this prevention. They prevented cesarean scar endometriosis by performing an intro-flexed suture of the uterine incision and by closing the visceral and parietal peritoneum [22]. Placing the specimen in an endo-bag and irrigating the port with saline will prevent the development of endometriosis at the port site [23]. Changing gloves before repairing an episiotomy scar and making sure the wound is free of decidua are both effective ways to reduce the risk of endometriosis developing in the scar tissue [24].

Limitation of the study

Though our study met the minimum required sample size, a multi-centric study with a larger sample size may yield better results. Our study didn’t estimate the recurrence rate since this was a cross-sectional study. Since this study was an exploratory study, we did not assign an outcome variable, and we performed no inferential statistics.

## Conclusions

We directly related surgical scar endometriosis to obstetrics and gynecological surgeries. Cesarean section is an obvious risk factor for surgical scar endometriosis. Clinicians should have a high index of suspicion for surgical scar endometriosis in women presenting with cyclic pain on the scar site. Ultrasound is accurate in diagnosing scar endometriosis. Medical management offers only temporary relief to be presented at a later date, and hence surgical intervention is the definitive treatment. Surgical management by wide local excision with a clear margin with or without mesh repair is the treatment of choice. We can prevent this iatrogenic complication by practicing certain surgical precautions.
